# Coping Strategies for Health and Daily-Life Stressors in Patients With Rheumatoid Arthritis, Ankylosing Spondylitis, and Gout: STROBE-Compliant Article

**DOI:** 10.1097/MD.0000000000000600

**Published:** 2015-03-13

**Authors:** Ingris Peláez-Ballestas, Annelis Boonen, Janitzia Vázquez-Mellado, Isabel Reyes-Lagunes, Adolfo Hernández-Garduño, Maria Victoria Goycochea, Ana G. Bernard-Medina, Jacqueline Rodríguez-Amado, Julio Casasola-Vargas, Mario A. Garza-Elizondo, Francisco J. Aceves, Clara Shumski, Ruben Burgos-Vargas

**Affiliations:** From the Department of Rheumatology (IP-B, JV-M, JC-V, RB-V), Hospital General de México “Eduardo Liceaga,” Mexico City, Mexico; Maastricht University Medical Center (AB), The Netherlands; Postgraduate Department of Psychology (I-RL), Universidad Nacional Autónoma de México, Mexico City; Department of Pediatrics (A-HG), Hospital Universitario “José Eleuterio Gonzalez”, Monterrey, Nuevo Leon; Clinical Epidemiology Unit (MVG), Hospital Gabriel Mancera Regional 1, IMSS, Mexico City; Department of Rheumatology (AGB-M), Hospital Civil de Guadalajara, Guadalajara, Jalisco; Department of Rheumatology (JR-A, MAG-E), Hospital Universitario “José Eleuterio Gonzalez,” Monterrey, Nuevo Leon; Hospital General de zona 46 (FJA), IMSS and Unidad de Investigación Crônico-Degenerativas, Guadalajara, Jalisco; and Department of Rheumatology (CS), Hospital Central, PEMEX, Mexico City, Mexico.

## Abstract

This article aims to identify the strategies for coping with health and daily-life stressors of Mexican patients with chronic rheumatic disease.

We analyzed the baseline data of a cohort of patients with rheumatoid arthritis (RA), ankylosing spondylitis (AS), and gout. Their strategies for coping were identified with a validated questionnaire. Comparisons between health and daily-life stressors and between the 3 clinical conditions were made. With regression analyses, we determined the contribution of individual, socioeconomic, educational, and health-related quality-of-life variables to health status and coping strategy.

We identified several predominant coping strategies in response to daily-life and health stressors in 261 patients with RA, 226 with AS, and 206 with gout. Evasive and reappraisal strategies were predominant when patients cope with health stressors; emotional/negative and evasive strategies predominated when coping with daily-life stressors. There was a significant association between the evasive pattern and the low short-form health survey (SF-36) scores and health stressors across the 3 diseases. Besides some differences between diagnoses, the most important finding was the predominance of the evasive strategy and its association with low SF-36 score and high level of pain in patients with gout.

Patients with rheumatic diseases cope in different ways when confronted with health and daily-life stressors. The strategy of coping differs across diagnoses; emotional/negative and evasive strategies are associated with poor health-related quality of life. The identification of the coping strategies could result in the design of psychosocial interventions to improve self-management.

## INTRODUCTION

The cognitive and behavioral adjustments that an individual uses to confront and manage health and daily-life stressors are referred to as “coping.”^1–4^ Strategies for coping arise from interpersonal components, the type of physical stressor, and sociocultural background.^1,5^ According to Folkman and Greer,^4^ and Sharpe and Curran,^5^ coping refers to the cognitive and behavioral adjustments made by the individual to confront and manage life stressors.^4,5^ Despite coping considered to be a personal trait, the individual may also develop specific strategies to confront stressors such as disease symptoms, functional limitations, psychological impact, and well-being low level.^1^ Although the spectrum of coping extends from the passive avoiding type to the active positive adjustment,^3^ the way of coping with chronic disease involves personal components, stressor's nature, and sociocultural background.^3,6^

Patient's beliefs and perception of illness influence the development of coping strategies. Despite coping being an individual trait, it might change over time, and in fact, most individuals develop specific strategies to confront specific stressors. The effect of coping strategies is usually classified as active or favorable, and passive or unfavorable.^1,2^ Of these, the “passive avoidant” and “active positive” strategies predominate in patients with stressful, chronic diseases; the former is also associated with diseases with the worst health outcomes.^1,2^

Rheumatoid arthritis (RA), ankylosing spondylitis (AS), and gout are painful and disabling chronic diseases that may profoundly affect quality of life of the patients and their relatives.^6^ The role of coping in patients with RA,^7–9^ as well as in patients with AS^10^ and other rheumatic diseases, has been studied.^11–13^

In general, the negative-emotional and evasive—passive/avoidant strategies—are risk factors for poor adjustments to chronic diseases and poor outcome of variables such as quality of life, pain, adherence to treatment, and risky behaviors.^3,6^ Yet, it is still unknown if the way in which a particular person confronts health stressors is similar to that when coping with daily-life stressors. Similarly, we do not know whether coping strategies differ across diseases and whether such variations result from differences in disease-associated stressors or differences in personality traits.^3,6^ As cultural background plays an important role in coping, the study of different populations could shed light on the way people confront health and daily-life stressors. Based on that information, we hypothesize that depending on the disease, mental and physical health components, as well as personal and cultural factors, the way people confront health differs from that confronting daily-life stressors. In addition, we hypothesize that the evasive and emotional/negative coping strategies are associated with poor health-related quality of life (HRQoL) whereas reappraisal and evasive strategies do not affect HRQoL.

Therefore, in this study, on one hand, we aimed to compare the strategies for coping with health stressors as well as daily-life stressors in Mexican patients with RA, AS, and gout, and, on the other hand, the effect of variables on coping and health status in each particular disease.

## METHODS

This article is a cross-sectional study of the baseline data of a cohort of patients with RA,^14^ AS,^15^ and gout^16^ that determined their socioeconomic impact in Mexico.^17^ The cohort consisted of 693 outpatients, with disease onset after the age of 18 years, attending 11 institutional and private centers in 5 major cities in Mexico. The Institutional Review Board, with all the following centers: Hospital General de México “Eduardo Liceaga,” Hospital Universitario “José Eleuterio Gonzalez,” Hospital Gabriel Mancera Regional 1-IMSS, Hospital Civil de Guadalajara, Hospital General de zona 46-IMSS, and Hospital Central PEMEX, approved the study's protocol and patients agreed to their participation in the study by signing an informed consent form.

Sociodemographic variables included sex, age, occupation, paid-job status, disability allowances, monthly family income, health resource utilization, and disease cost impact (1 = no impact, 2 = moderate impact, 3 = high impact). Clinical variables included those obtained by clinical history and physical examination, as well as pain level with a numerical rating scale (NRS; 0 no pain, 10 unbearable pain), and health status by the short-form (SF-36) questionnaire.^18^ In addition, patients with RA and gout filled the Health Assessment Questionnaire (HAQ),^19^ and patients with AS completed the Bath AS Disease Activity Index (BASDAI),^20^ the Bath AS Functional Index (BASFI), and the Bath AS Global Well-being (BASG) Indexes.^21^ The Cronbach α was estimated for all measurements made.

The type of coping and its characteristics were assessed with a self-administered questionnaire developed and validated by Góngora^3^ in the Mexican population, following Folkman and Greer theory.^4^ Briefly, the questionnaire assessed the 2 domains of coping, health and daily life. Each of these domains included 18 questions related to 4 primary coping strategies: direct strategy, in which the individual attempted to adjust cognitively or behaviorally tackle the problem (eg, “When I have health problems, I take care of myself by following a course of treatment”); emotional strategy, in which the individual deals with a problem in an emotional or negative way and expresses feelings that do not solve the problem directly (eg, “When I have health problems, I get upset”); evasive strategy, in which the individual is willing to escape, avoid, or minimize the problem (eg, “When I have health problems, I try to sleep because I do not want to think about it”); and reappraisal strategy, in which the individual tries to deal with the problem positively or somehow improves his/her perception of it (eg, “When I have health problems, I realize how important life is”).^3^ Each of the 4 coping strategies is covered by 3 to 5 questions, providing a total of 18 for each of the domains. The response to each question is scored in a 7-point NRS, anchored with the levels “never” (1) and “always” (7). The scoring system includes the calculation of the mean of the answer given to each of the 4 coping strategy questions pertaining to health and daily-life domains. Based on the results of healthy individuals, mean values ≥4 indicate the dominance of ≥1 coping strategies in a particular patient.^3^

The sample size was obtained assuming a prevalence of 1% reported elsewhere, with a confidence level of 95% and the margin of error of ±0.05, producing a total of 136 patients. Given that there were 5 referring institutions, 200 individuals were considered. Taking into account a 15% follow-up loss, the total of patients considered for the study was 224 in each group of disease, with a statistical power of 0.74. Sampling was nonprobabilistic. Questionnaire with missing data was excluded.

### Statistical Analyses

Sociodemographic, clinical measures and coping strategies were reported using descriptive statistics for each of the 3 disease groups and for the whole group of patients that included the Kruskal–Wallis test, ANOVA with Bonferroni's correction, and the χ^2^ test for continuous and categorical variables across diseases with a statistical significance level of 0.05 (2 sides). Results are expressed as odds ratio and 95% confidence intervals. Analysis included the whole patient population and each of the 3 diagnostic groups.

The variables included in the models had a statistical significance of at least 0.2 and biological plausibility in the univariate analysis. The relation between coping strategies and health status (Physical Component Scale [PCS] and mental component Mental Component Scale [MCS] of short form [SF]-36) was analyzed in 2 linear regression models. In the first model, age, sex, economic impact, social support, and coping mechanisms were independent variables, whereas health status was dependent variable. In the second model, coping was the dependent variable whereas independent variables were age, sex, economic impact, social support, and health status.

To explore the influence of coping on physical and mental health, 4 simple and 3 multiple regression models were performed with PCS and MCS-SF-36 components as dependent variables. The first model combined the 3 diagnostic groups. The next 3 models corresponded each to 1 diagnostic category. The 4 regression models shared first and second blocks of independent variables. The third block differed across regression models. In the first model, we included those clinical variables shared by all diagnostic categories, specifically disease duration and pain severity. The third block also included disease duration and pain severity as well as HAQ and Diseases Activity Scale (DAS28) for the RA model; BASFI, BASDAI, and BASGI for AS; and swollen joint count, tophi count, visual analog scale general health, and HAQ for gout. Interactions between coping strategies and disease toward each of the components of SF-36 were sought. Collinearity between variables was evaluated using 0.9 as threshold for acceptability. The models were evaluated using goodness-of-fit that was performed using the Hosmer–Lemeshow test. Interactions between coping mechanisms and disease toward each of the components of HRQoL were sought. Analyses were performed using STATA 9.0 statistical software (StataCorp LP, College Station, TX).

## RESULTS

In total, 693 participants were included in the study; their mean age (standard deviation) was 45.1 (14.8) years; 371 (53.5%) were men; 261 (37%) had RA, 226 (32.5%) had AS, and 206 (29.6%) had gout (Table 1). Sociodemographic variables differed across diagnoses. PCS-SF-36 and MCS-SF-36 scores in patients with RA and AS were comparable and lower in patients with gout.

**TABLE 1:**
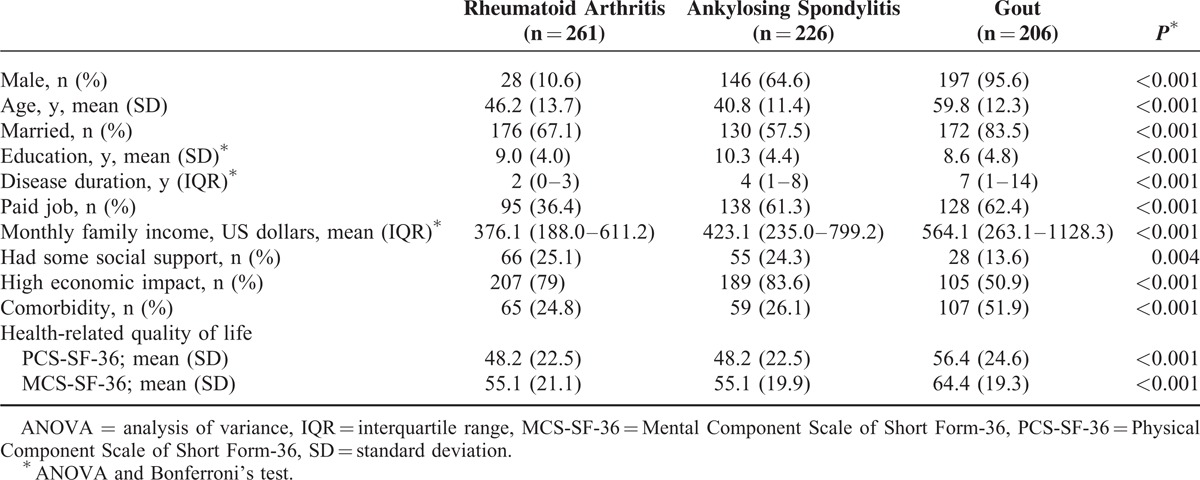
Sociodemographic and SF-36 Features of Patients Included in the Study

**FIGURE 1:**
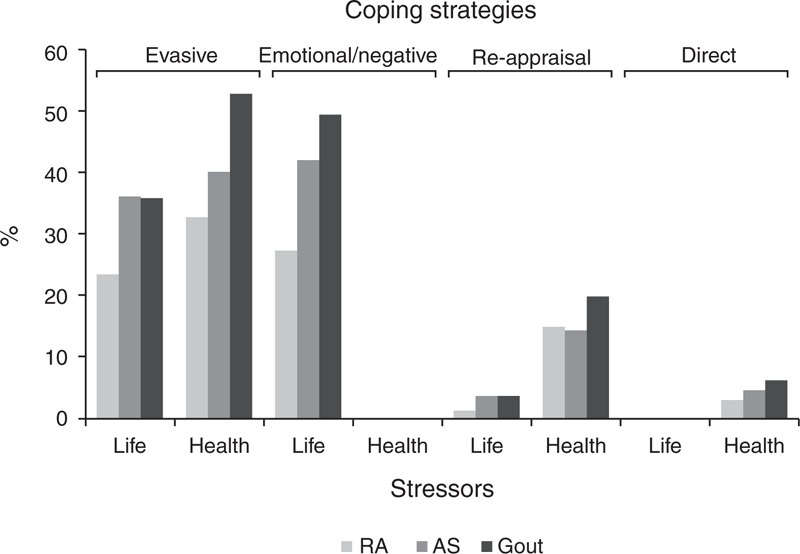
Frequency of coping strategies in patients with RA, AS, and gout. The frequency of each of the 4 coping strategies is presented in 2 blocks, daily health and daily-life stressors. The proportion of patients with RA, AS, and gout using the evasive strategy to cope with health and daily-life stressors was higher than those using other strategies. The emotional/negative strategy was particularly used when coping with daily life, but not when coping with health stressors. Reappraisal was used by a small proportion of patients coping with both health and daily-life stressors. Few patients used a direct strategy to cope with health, but no daily-life stressors. Statistical analysis (ANOVA–Bonferroni test) showed significant differences between groups regarding the use of the evasive strategy to cope with health and daily-life stressors (*P* = 0.000 and 0.003, respectively) and the emotional/negative strategy to cope with daily-life stressors (*P* = 0.000). ANOVA = analysis of variance, AS = ankylosing spondylitis, RA = rheumatoid arthritis.

Three hundred sixty-seven (53%) patients and 326 (47%) patients had ≥1 coping strategy to confront health and daily-life stressors, respectively (Figure 1). Men confronted health stressors with the evasive strategy (59.1% vs 30.9% in women, *P* ≤ 0.001), and daily-life stressors with the emotional/negative (50.6% vs 25% in women, *P* ≤ 0.001) and evasive strategies (37.2% vs 24.7% in women, *P* ≤ 0.001) (Table 2).

**TABLE 2:**
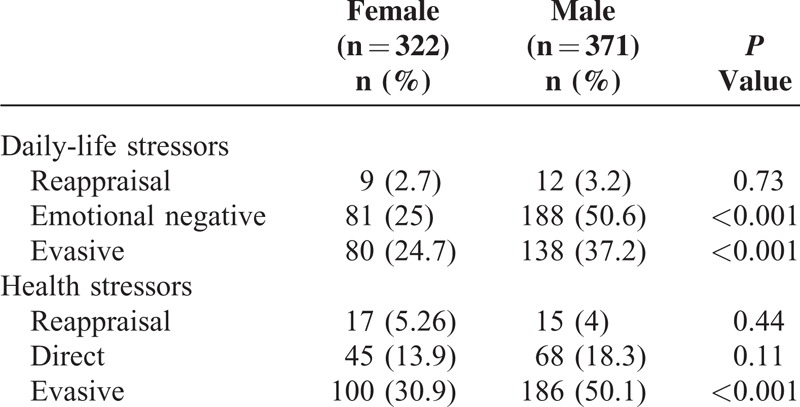
Strategies for Coping With Health and Daily-Life Stressors According to Sex

Few patients confronted health stressors with direct strategies, or daily-life stressors with reappraisal. Evasive and reappraisal strategies prevailed when patients coped with health stressors, whereas emotional/negative and evasive strategies predominated in patients coping with daily-life stressors (Figure 1) (Table 3). Overall, the proportion of patients coping with specific health and daily-life stressors differed across diagnoses. More patients with gout relied on coping strategies than patients with AS and RA. Reappraisal, emotional/negative, and evasive strategies were prominent in patients with gout and AS.

**TABLE 3:**
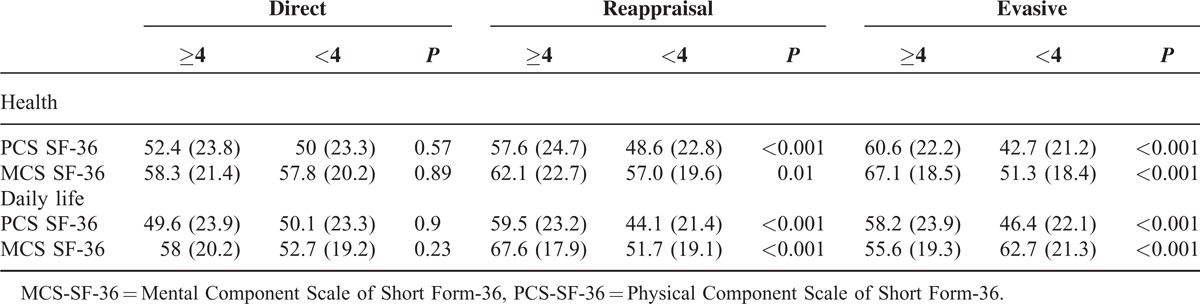
Strategies for Coping With Health and Daily-Life Stressors According to Health-Related Quality of Life

The distribution of coping patterns across life and health dimensions in the 3 diseases was different. Reappraisal together with evasive and direct strategies was the most common coping pattern for health dimension (Table 3).

The best-fitting models to explain the influence of sociodemographic and clinical variables on the use of evasive and direct strategies to cope with health stressors were fairly similar across diagnoses, including the type of disease used as dummy variable in the multivariate analysis (Table 4). The evasive coping strategy was most likely to be used by patients having RA with higher MCS-SF-36 scores and older age, and by patients with AS and gout with higher MCS-SF-36 and PCS-SF-36 scores. In patients with gout, the use of direct coping strategies was associated with a high economic impact. When the 3 diagnostic categories were combined, PCS-SF-36 scores explained the evasive and direct strategies and MCS-SF-36 scores the evasive strategy. In contrast, being female and having a paid job eliminated the use of the evasive strategy.

**TABLE 4:**
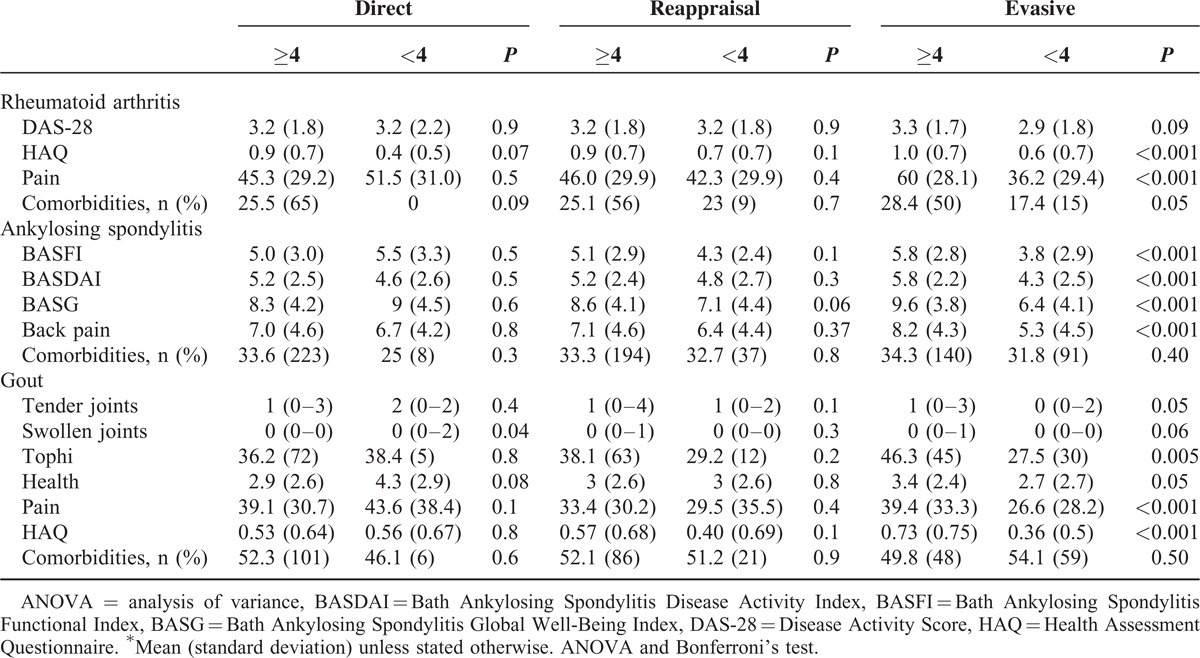
Strategies for Coping With Health Stressors Across Diagnoses and According to Disease Outcomes^∗^

Patients using the evasive strategy for coping with health stressors were more likely to have low PCS-SF-36 and MC-SF-36 scores, regardless of their disease (Table 5). In addition, high HAQ scores in patients with RA and gout, as well as high BASFI and BASDAI scores in AS, were associated with low PCS-SF-36 and MCS-SF-36 scores. High HAQ scores and the use of the evasive coping strategy were associated with low SF-36 scores and high levels of pain with low PCS-SF-36 scores in patients with gout.

**TABLE 5:**
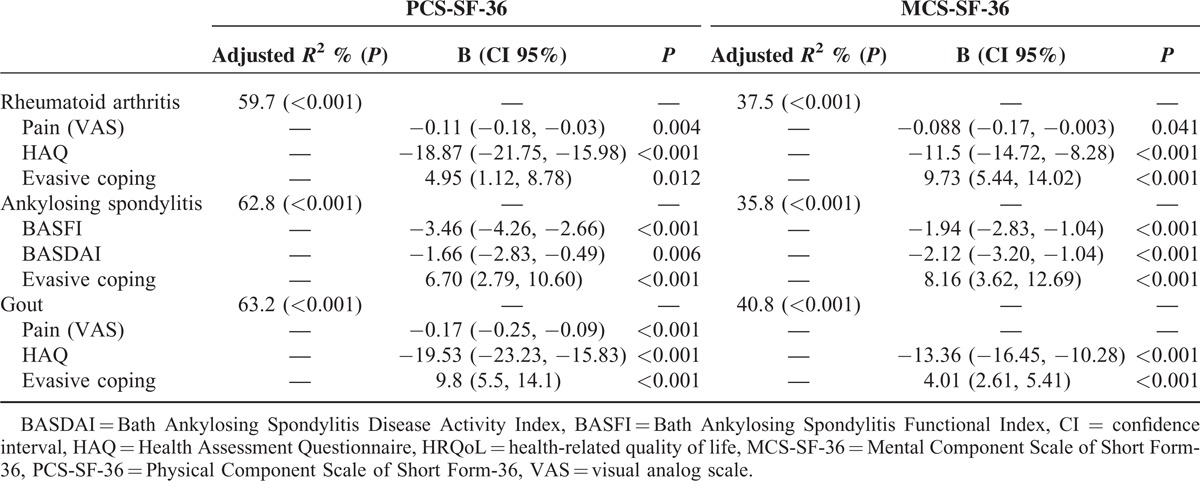
Multiple Linear Regression Models to Explain the Role of Coping Strategies With Health Stressors and Clinical Variables According to Diagnostic Categories on HRQoL as Measured by SF-36

The Cronbach α estimated for the internal consistency of each measure was 0. 85 (SF-36), 0.87 (BASDAI), 0.95 (BASFI), 0.96 (HAQ), and 0.85 (coping scale). With the exception of “direct” health coping style (0.27), the statistical power for all measurements was between 0.78 and 0.99.

## DISCUSSION

In this study, we found that the strategies of rheumatic patients with RA, AS, and gout to cope with daily-life stressors differ from those to cope with health stressors. On the other hand, we found differences in coping strategies across diagnosis. These findings suggest that coping is a complex phenomenon, in which the type of stressor and the patient's diagnosis influence the way in which the individual confronts health and daily-life problems.

Regarding daily-life stressors, most patients showed the emotional or negative coping strategy, but regarding health stressors, none of the diagnostic categories relied on such strategy. Instead, health stressors were confronted with the evasive, reappraisal, or direct coping strategies. These findings differ from the predominant reappraisal and then evasive strategies used by Mexican individuals without chronic disease to cope with health stressors.^3^ Two factors, chronicity of stressors and the predominance of musculoskeletal symptoms, appear to influence the mechanisms of coping toward specific strategies. In addition, low educational level, poor health outcomes, and individual's independent social role^22^ have been associated with emotional/negative and evasive coping strategies. On the contrary, the reappraisal strategy has been associated with better outcomes.

Interestingly, the strategies for coping with health stressors in patients with gout were those associated with low compliance and poor health status. In the multivariate analyses, patients with gout and AS—who were mainly men—relied more frequently on the emotional/negative and evasive coping strategies. Until now, there was no information about the use of such strategies by men with musculoskeletal disease who confronted health stressors. Interestingly, women without chronic disease rely in the same strategies^3^ as women with depressive illness.^23^ In this study, the strategies of women, particularly with RA were similar—but slightly less frequently—to those found in men with gout and AS. Despite this evidence, sex did not explain the relationship between coping patterns and health status in the multivariate analysis.^7–9^

In healthy Mexican controls, the reappraisal and then evasive strategies were the most frequently used to cope with health and daily-life stressors.^3^ The sequence in our patients with RA, AS, and gout was quite different: the predominant strategy was evasive, whereas only a small proportion of patients relied on reappraisal. These findings suggest that the predominance of coping strategies in patients facing chronic disease turns from a positive pattern, specifically reappraisal, to a negative pattern, which in this study consisted mainly of evasion.

The use of evasive strategies for coping reflects the poor adjustment to disease of non-Western cultures including the mix of Amerindians and European that populates most parts of the American continent.^1^ Such type of adjustment has been associated with low health status and poor disease conditions, including high level of pain, low adherence to treatment, and long-term risk behavior in patients with chronic diseases,^1,5,24–26^ RA,^7–9^ AS,^10^ polyarthritis,^12^ systemic lupus erythematosus,^11^ and juvenile fibromyalgia.^13^ On the contrary, Western cultures rely on direct and reappraisal strategies rather than in the evasive/emotional for coping with health stressors.^1,22^ In contrast to evasive coping, direct and reappraisal strategies are associated with less pain and fatigue, low HAQ score, and better physical and mental status.^8,9^ In addition, the use of coping strategies for better adaptation, for example, optimism and “comforting cognition,” has been associated with high levels of health status.^2,8,9,22^ Patients using direct coping generally have good social as well as family relationships, good educational level, and stable jobs.^8,9,26^

In agreement with other studies,^5,7–9^ we also found an association between health status and specific clinical variables. Thus, high DAS28, HAQ, BASDAI, and BASFI scores were associated with low PCS-SF-36 and MCS-SF-36 scores. In addition, lower rates of pain and physical limitations were associated with lower health status. Studies suggest that evasive coping mediates disease representation, clinical variables, and disease outcomes, such as disability, health status, and psychiatric comorbidity,^1,8,9,23^ but data are still inconclusive.

Based on the results of this study, we propose that contextual variables, such as cultural characteristics, influence coping strategies. This position would help to clarify the indirect relationship between coping patterns, outcomes, and treatment decisions. As mentioned elsewhere, a limitation for the investigation of coping and the adjustment to chronic disease is related to the scant attention given to the cultural aspect, ethnic identity, acculturation processes, and socioeconomic status of the target population.^1,3^ This study offers a possible explanation of the differences in coping processes in Mexican patients with rheumatic disease representing an ethnically heterogeneous population, in which cultural variables play an important role. The difference in the response rate of some variables found in some multinational clinical trials could be associated with geographic and cultural differences.^11,25,26^ Ethnicity is an adjustment predictor for chronic diseases, for example, cancer in African Americans and Hispanics.^5^ Taking altogether, the information provided in this article and that from the literature supports the need for a better physician–patient (Hispanic in this case) relationship and the possibility of developing well-oriented programs of health care for the community. In this sense, our findings provide the basis for a more direct approach of Hispanic patients in countries with various health care systems, including the United States in North America and the European Community. It is clear that the contribution of cultural differences to mental and physical health status should be explored in future studies.

The most important characteristic of the collectivistic pattern of culture, which predominates in Latin American, African, and some Asian groups, is the interdependence of the cultural group.^22,23–25^ In contrast, the individualistic cultural pattern, which is shared by most Western cultures, is characterized by the independence of the individual.^25^ The collectivist and individualistic culture patterns may cause differences in the relationship between coping and health status and explain one of the key findings of our study, the predominant use of the evasive strategy for coping and its association with good health status and disease status. This finding contrasts with the strategies of healthy Mexican individuals when confront a nonchronic disease, and the strategies of coping found in healthy and chronically diseased individuals in Western cultures.

In addition, Triandis^25^ has shown that the way people cope with life stressors is highly dependent on the population characteristics. In individualistic societies, coping strategies differ from those showed in collectivistic societies. Coping is a trait that mediates stress and the mechanisms involved in physical and mental adaptation.^3,4^ Diaz-Guerrero^27,28^ proposal of a sociocultural control of stress refers to the existence of an active/passive dichotomy. The strategy to cope with stress that predominates in Mexicans and, in this study, in patients with gout is passive and includes the evasive and emotional profiles.^25,27^ In contrast, the strategies for coping that predominate in the United States are direct and reappraisal. Although Mexican society is collectivistic, the American is individualistic.

As the results of our study differ from those found in rheumatic patients from individualistic societies, we proposed that rather than considering any strategy better or worse than another, it is important to identify the social environment in which the individual is inserted and adjust the way he copes to improve his quality of life.

We acknowledge that the cross-sectional design of this study prevents the identification of causal inferences and therefore the effect of variables in coping patterns. However, we identified a number of variables significantly associated with independent variables, for example, the association between coping patterns and SF-36, which influence the development of interventional strategies for coping modulation. The fact that we did not focus on psychopathology, patient perception of the disease, and spirituality may be also a limitation in our study, but the assessment of these domains require specific research projects. The fact that the questionnaire on coping was specifically developed in Mexico might be seen as a limitation in our study, but despite the idea that instruments, including patient-oriented questionnaires, should be universal and reproducible across countries, we consider that coping with health and daily-life stressors is strongly associated with sociocultural factors. In this regard, the use of negative strategies when dealing with stressful daily-life events, particularly in males, could be partially explained by the collectivist culture pattern of the Mexican society.^3^ In this sense, the indirect comparison of studies carried out across different populations may also provide information on the role of sociocultural factors in each population.

In conclusion, patients with some chronic rheumatic diseases develop coping strategies to confront health stressors, which differ from those used to confront daily-life problems. On the other hand, the predominance of coping strategies varies across diagnoses and sex. Patients with gout relied more often on coping strategies than those with RA or AS, after correcting for other confounders. The negative strategy for coping with health stressors is associated with poor physical and mental health outcomes. It is important to consider that the interpretation of these findings should be made in the context of specific culture. On the contrary, in clinical practice, it is equally important to develop treatment strategies according to coping patterns as an important variable in the development of the disease.
